# Expertise, Automation and Trust in X-Ray Screening of Cabin Baggage

**DOI:** 10.3389/fpsyg.2019.00256

**Published:** 2019-02-14

**Authors:** Alain Chavaillaz, Adrian Schwaninger, Stefan Michel, Juergen Sauer

**Affiliations:** ^1^Department of Psychology, University of Fribourg, Fribourg, Switzerland; ^2^School of Applied Psychology, University of Applied Sciences and Arts Northwestern Switzerland, Olten, Switzerland

**Keywords:** airport security, expertise, trust, automation, detection performance

## Abstract

X-ray screening of passenger baggage is a key component in aviation security. The current study investigated how experts and novices performed in an X-ray baggage screening task while being assisted by an adaptable diagnostic aid. Furthermore, it examined how both groups operated and trusted this automated system. 30 experts (certified screeners) and 31 novices (students) had to indicate whether a target item (either a knife or a gun) was present in a series of X-ray images of cabin baggage. Half of the participants could choose between three different support levels of the diagnostic aid (DA): (1) no support, (2) a cue indicating the presence of a potential target without locating it, or (3) a cue indicating the presence of a potential target by surrounding it with a red frame. As expected, experts achieved higher detection performance (d’), were more self-confident and felt more competent in achieving the task than novices. Furthermore, experts experienced less time pressure and fatigue. Although both groups used the DA in a comparable way (in terms of support level used and frequency of level switches), results showed a performance increase for novices working with the DA compared to novices without support. This benefit of DA was not observed for experts. Interestingly, despite no difference in perceived trust ratings, experts were more compliant (i.e., following DA recommendations when it indicated the presence of a target) and reliant (i.e., following DA recommendations when it indicated the absence of a target) than novices. Altogether, the results of the present study suggested that novices benefited more from a DA than experts. Furthermore, compliance and reliance on DA seemed to depend on expertise with the task. Since experts should be better at assessing the reliability of the DA than novices, they may have used the DA as ‘back-up’ to confirm their decisions based on expertise (confirmatory function), while novices may have used it as a guide to base their decisions on (support function). Finally, trust towards a DA was associated with the degree to which participants found the DA useful.

## Introduction

Secure air transportation is crucial for economy and society because airplanes have been valuable targets for terrorists for several decades (Abadie and Gardeazabal, 2008; Baum, 2016). X-ray screening of passenger bags at airport security checkpoints is a key component of aviation security measures to ensure that prohibited items cannot be brought on airplanes (Harris, 2002). In recent years, automated explosive detection has become available for cabin baggage screening (Sterchi and Schwaninger, 2015). Such systems indicate the potential presence of explosive material in X-ray images of passenger bags to assist airport security officers (screeners) in visual search and decision making (Hättenschwiler et al., 2018). This corresponds to diagnostic automation, a type of low-level automation providing support in the form of alerts or alarms (Wickens and Dixon, 2007; Cullen et al., 2013). In addition to automated explosive detection, algorithms for detecting guns and knives have been developed in recent years (e.g., Mery et al., 2013) and such systems are currently being tested at airports (Lehr, 2019). While a substantial amount of research is now available on visual inspection and visual search in X-ray images of passengers bags (see Biggs et al., 2018 for a recent review), only few studies have been conducted on automation as diagnostic aid in X-ray baggage screening (Wiegmann et al., 2006; Chavaillaz et al., 2018; Hättenschwiler et al., 2018). The relationship between automation, expertise and different aspects of trust in X-ray baggage screening has not been investigated yet.

To address this research gap, we conducted a study using a baggage screening task. Students and screeners that were tested with and without automation as diagnostic aid to examine its impact on detection performance, trust perceptions, trust intentions, and trust behaviors. In addition, self-confidence and perceived workload were measured. In the remainder of this introduction, we first summarize previous research on visual inspection and expertise in X-ray baggage screening. We then discuss automation and several aspects of trust before we finish with the research questions of this study.

### Visual Inspection and Expertise in X-ray Baggage Screening

During screening at airport security checkpoints, screeners visually inspect X-ray images of passenger bags to decide whether they are harmless or whether they might contain a prohibited item and therefore require secondary search (typically using explosive trace detection and manual bag search; Sterchi and Schwaninger, 2015). Examples of prohibited items are guns, knives, improvised explosive devices, self-defense gas sprays or electric shock devices (Schwaninger, 2005). X-ray image inspection by airport security officers (screeners) involves visual search and decision making (Koller et al., 2009; Wales et al., 2009; Wolfe and van Wert, 2010). Visual search challenges include a low target prevalence, the variation in target visibility, the search for an unknown target set, and the possible presence of multiple targets (for recent reviews, see Biggs and Mitroff, 2014; Mitroff et al., 2015; Biggs et al., 2018).

Visual expertise reflects complex cognitive and perceptual processing. It develops over the course of many hours of practice and training (for reviews, see Dzindolet et al., 2003; Ericsson et al., 2006; Reingold and Sheridan, 2011; Sheridan and Reingold, 2017). This is of particular importance for X-ray image inspection, because in X-ray images, many objects look very different than from reality (Schwaninger et al., 2005). Several studies have shown that initial and recurrent computer-based training is necessary to achieve and maintain high detection performance in visual inspection of X-ray images of passenger bags (Schwaninger, 2005; Koller et al., 2008, 2009; Halbherr et al., 2013; Schuster et al., 2013). International regulations take this into account by mandating initial and recurrent training of screeners. For example, European regulations require at least 6 h of image recognition training and testing in every 6-month period for X-ray screeners at airports (European Commission, 2015). In a study conducted with 5717 aviation security screeners over a period of four years, Halbherr et al. (2013) showed that the relationship between the amount of computer-based training and gains in detection performance follows a logarithmic function with large initial gains up until around 50 h. Research in medical image inspection has repeatedly shown that experts have a much higher detection performance than novices while at the same time experts need less time for visual search and decision making (for recent reviews see Blondon et al., 2015; Wolfe et al., 2016; Litchfield and Donovan, 2017). As discussed in detail by Sheridan and Reingold (2017) such effects of expertise could be related to holistic processing, which allows more efficient and effective visual inspection. Consistent with these findings, Koller et al. (2009) found that screeners had faster search and decision times than novices, when visually inspecting X-ray images of cabin baggage. This could result in experts’ feeling more self-confident but also in experiencing less workload than novices, an issue that we wished to address in our study as well.

### Automation and Trust

As explained above, achieving the expertise level of a certified screener requires a long process, but it may be accelerated by automation (e.g., Hoffman et al., 2014; Jipp, 2016). Current technological developments allow for instance the automatic detection of guns (Mery et al., 2013) or explosive detection systems for cabin baggage screening (Wells and Bradley, 2012; Hättenschwiler et al., 2018). Recent studies showed the benefits of implementing automated detection in airport screening, as observed in other work domains such as process control (e.g., Rovira et al., 2007; Chavaillaz et al., 2016). Higher performance levels are achieved by experts in detecting explosives (Hättenschwiler et al., 2018) and by untrained participants in detecting hand guns and knives in cabin baggage (Chavaillaz et al., 2018).

To obtain such benefits, the human agent has to make good use of automation (Zuboff, 1998). One factor influencing automation use is how much trust is placed in automation (Lee and See, 2004). Past experiences of an operator with the technology can influence how he or she trusts that the machine will behave similar in other situations. Furthermore, experts can calibrate trust because of sufficient experience with the technology (Hoffman et al., 2013). Trust can take three facets, perception, intention and behavior (Lee and See, 2004). Trust perception reflects the trustworthiness of the system. Trust intention corresponds to the willingness to rely on the system, while trust behavior is the actual compliance or reliance on the system. According to Meyer (2001), users show compliance towards the DA when they follow its recommendations when it triggers an alert. On the other hand, reliance corresponds to user’s propensity to acknowledge DA recommendations when it indicates having detected no issue. Many studies investigated those aspects in the context of support systems (e.g., Dzindolet et al., 2003; Rovira et al., 2007; Chavaillaz et al., 2018), but it seems that no research considered the impact of expertise in an inspection task. The current work addressed this issue for trust perception and behaviors.

Most recent X-ray machines (e.g., Lehr, 2019) provide an automatic support system (also referred to as diagnostic aid, or DA) where a potential target is surrounded by a red rectangle. In contrast, during computer-based training, screeners typically receive no automatic support when learning to recognize and detect threat items in passenger bags. The support system can be compared to the levels of automation (LOA) based on the work of Goh et al. (2005). All three levels of automation have their advantages and disadvantages and therefore to investigate the usefulness of each system for novices and experts is of scientific interest. LOA 3 for example (where a direct cue is provided) could lead to missing other threat items, which are not marked (i.e., influence on the quitting threshold in a multiple-target search task; for a review, see Biggs, 2017). LOA 1, where no support is provided, could increase cognitive workload and fatigue (e.g., Parasuraman and Hancock, 2008). LOA 2, where an indirect cue is provided by surrounding the whole piece of luggage, could therefore reduce the disadvantages of LOA 1 and 3.

### Present Study

The current study examined how expertise and automation influence visual inspection performance, as well as automation use and trust in automation. Certified screeners and students were instructed to indicate whether a prohibited object (i.e., a knife or a gun) was present in a series of gray-scaled X-ray images of baggage. Half of both groups were assisted by an automated diagnostic aid, while the other half completed the task without support. This system had three levels among which participants could freely choose at any time during the whole experiment: (1) no cue, (2) an indirect cue indicating the presence (or absence) of a potential target, and (3) a direct cue surrounding the target with a frame.

We expected that experts will show higher detection performance (d’) and shorter response times than novices (e.g., Michel et al., 2007), as well as higher ratings of self-confidence and competence (Shanteau, 1992). Furthermore, they will experience less fatigue and time pressure. Regarding the diagnostic aid, novices will benefit more from the diagnostic aid than experts because they have almost no experience in the task and do not know which threat items are prohibited and what they look like in X-ray images (Hättenschwiler et al., 2018). Finally, novices will be more compliant and reliant than experts.

## Materials and Methods

### Participants

30 certified screeners (14 females) and 31 students (24 females) were tested. All screeners had been selected, qualified, trained, and certified according to the standards set by the appropriate national authority (civil aviation administration) in compliance with the relevant EU regulation (European Commission, 2015). They were aged between 24 and 60 years (*M* = 45.64, *SD* = 8.93) and had at most 36 months of experience in baggage screening (*M* = 16.51, *SD* = 10.08). Students were aged between 18 and 54 years (*M* = 26.27, *SD* = 6.53) and had no prior experience in baggage screening. All participants had normal vision or corrected to normal with glasses or contact lenses. The Ethics Committee of the Department of Psychology at the University of Fribourg (Switzerland) gave their approval for this study. Informed consent was obtained from all participants.

### Design

The current study used a 2 × 2-factorial design, with expertise and diagnostic aid as between-subjects factors. Regarding expertise, half of the participants were certified screeners, while the other half of the participants was novices (see section ‘Participants’ for more details). Regarding the diagnostic aid, half of the participants worked with a support system, whereas the other half did not. Experts and novices were randomly assigned to one of the diagnostic aid conditions.

### Apparatus and Stimuli

The baggage screening simulation was controlled by an Octave script using the Psychtoolbox (Brainard, 1997; Pelli, 1997; Kleiner et al., 2007). A Dell laptop with Windows 10 as operating system was used to present the X-ray images. The screen had a resolution of 1920 × 1080 pixels and a refresh rate of 60 Hz. Participants sat a in a very quiet room with constant dim light at an approximate distance of 0.70 m from the screen and could freely move their head. Displayed X-ray images of baggage covered a maximum surface of 13.76 × 11.90 deg of visual angle.

Stimuli for the pretest came from the X-ray Object Recognition Test (X-Ray ORT, Hardmeier et al., 2005; Schwaninger et al., 2005). Stimuli for the main experiment came from a newer version of the X-ray ORT and from a competency assessment test (X-Ray CAT), which is used for screener certification. Threat items were restricted to guns and knives. Compared to explosives or electronic shock devices, their shapes are more familiar to novices from every-day life or every-day multimedia entertainment. Images were presented in grayscale since novices do not know the meaning of colors in X-ray baggage images.

### Simulation

**FIGURE 1:**
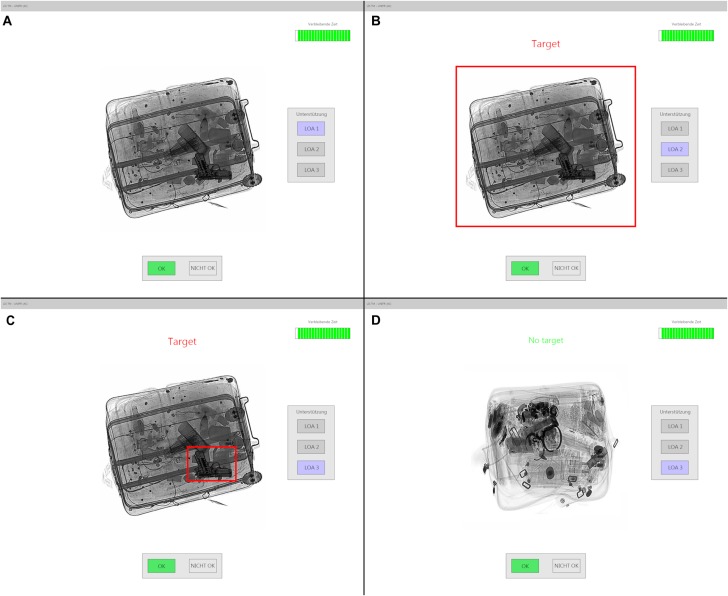
Interface of Luggage Inspection Simulation (LIS) depicting the levels of automations (LOA): **(A)** trial without support (LOA 1), **(B)** indirect cue (LOA 2), **(C)** direct cue (LOA 3), and **(D)** valid cue for a target-absent trial (LOA 3).

A modified version of the Luggage Inspection Simulation (LIS) served as model for the inspection task (see for instance, Chavaillaz et al., 2018). In this task, participants had to decide as fast and as accurately as possible whether each X-ray image contained a prohibited item (either a gun or a knife) or not (i.e. yes-no task in signal detection theory; Macmillan and Creelman, 2005). If no target was found in the image, they were instructed to click on the ‘OK’ button (see Figure 1). If they found a potential target, they had first to mark it (by clicking on it) and then click on the ‘Not OK’ button. This button remained inactive until participants marked an item in the X-ray image. Half of the images contained a target. The target-present/target-absent ratio was based on previous work. A change in this ratio will influence participants’ response bias but not their detection performance (Wolfe et al., 2007).

Each trial started with a fixation cross for 500 ms in the center of a white screen, followed by an X-ray image. The image disappeared when participants responded by clicking on the ‘OK’ or ‘Not OK’ button. If no response was provided within a time frame of 20 s, the trial stopped and was scored as a target-absence response. A blank screen was displayed for 500 ms between trials. In each trial, participants were informed of the remaining time by a bar countdown timer as showed in Figure 1 (i.e., the number of vertical bars corresponded to the number of seconds left to respond).

Like for the most recent X-ray machines (e.g., Lehr, 2019), LIS provided an automatic support system (also referred to as diagnostic aid, or DA) with three levels of automation (LOA) to assist participants in their task. This system was based on the work of Goh et al. (2005). At LOA 1, no support is provided. At LOA 2, when the support system detected a potential target (i.e., a gun or a knife), it provided an indirect cue (i.e., the whole piece of baggage was surrounded by a red frame with the warning ‘Target’ being written above the X-ray image, see Figure 1). At LOA 3, it provided a direct cue which pointed at the exact location of the potential target. If no target was detected, the message ‘No target’ was displayed at LOA 2 and 3. Participants could freely change the LOA as many times they wished when an X-ray image was displayed. The first trial of a session started at LOA 1 and the following trials started with the last LOA used in the previous trial. LOA 1 represents a typical computer-based training setting whereby LOA 3 represents the work environment at most international airports. LOA 2 has no actual link to screener’s training or work environment but corresponds to an intermediate level between LOA 1 and 3. It allows screeners to get some support while still providing some challenge and the freedom of an unbiased visual analysis. The reliability level of the support system was 75%. The system never missed a target (i.e., missed to report the presence of a target). Two types of failure could occur, false alarms and miscues. In the former case, the support system indicated the presence of a target when there is no target in the image. In the latter case, it cued a non-target item even though there was a target in the image. The same amount of failure types occurred during each experimental block. Participants were only informed that the support system might sometimes fail. The nature of the failures was not mentioned.

### Dependent Variables

#### Performance

Three measures assessed participant performance. *Detection performance* (i.e., participant ability to indicate the presence or absence of a target in X-ray images) was measured by d’ Green and Swets (1966). It is computed by the following formula = *z*(H) – *z*(FA). H refers to hit rate, FA to false alarm rate of participants, and *z* to the inverse of the cumulative distribution function of the standard normal distribution. *Response bias* corresponded to participant tendency to respond ‘yes’ or ‘no’ and measured by *c* = -0.5 ^∗^ [*z*(H) + *z*(FA)]. For more information on these measures see Green and Swets (1966) and Macmillan and Creelman (2005). Finally, *target localization* referred to the percentage of target correctly marked on the images.

#### Use of Diagnostic Aid

The *median LOA* quantified the degree of assistance required by participants. The number of *LOA switches* per trial referred to the stability of LOA selection.

#### Subjective State

A purpose-built item adapted from Lee and Moray (1992) measured participants’ *self-confidence* in their ability to perform the task: ‘How confident were you in your ability to detect prohibited items?’. It was rated on a 10-point Likert scale (ranging from ‘not at all’ to ‘completely’).

The NASA-TLX (Hart and Staveland, 1988) was used to assess *subjective workload*. Participants had to rate six items on a 20-point Likert scale (ranging from ‘not at all’ to ‘extremely’). Each item covered a specific dimension of workload (i.e., mental demands, physical demands, temporal demand, performance, frustration, and effort).

#### Trust

*Trust perception* was assessed by the 12-item questionnaire *Checklist of Trust between People and Automation* (CTPA; Jian et al., 2000). Answers were given on a 7-point Likert scale (ranging from ‘not at all’ to ‘totally agree’). An item example was ‘The system is reliable’.

Trust behaviors were measured by two measures (based on Meyer, 2001), both expressed as a percentage (see for instance Rice and McCarley, 2011). *Compliance* refers to participants’ tendency to confirm the presence of a target when the diagnostic aid said so. The following formula was used to compute compliance: TP_P_/TP_DA_. TP_DA_ corresponds to the number of trials for which the DA indicated the presence of a target, while TP_P_ refers to the number of trials for which participants responded “Bag not OK” when the DA indicated the presence of a target. On the other hand, *reliance* corresponds to participant’s inclination to approve the suggestions of the support system when it reported the absence of a target. It was computed as following: TA_P_/TA_DA_. TA_DA_ corresponds to the number of trials for which the DA indicated the absence of a target, while TA_P_ refers to the number of trials for which participants responded “Bag OK” when the DA reported the absence of a target in the X-ray image.

### Procedure

The experiment was composed of two distinct parts (pre-test and main test). The pre-test fulfilled two purposes. First, it was to ensure that certified screeners showed better detection performance than novices. Second, it was to control for possible differences in detection ability between participants working with and without diagnostic aid within each expertise level. The main test aimed to evaluate the impact of both expertise and the presence of the diagnostic aid on the outcome variables. Overall, participants needed about 60 min to complete the entire experiment.

The pre-test started with instructions describing the task and the response modalities. In contrast to the main test, the response buttons were not displayed on screen. Participants had to click on either the left or the right mouse button to provide a response. The stimulus-response mapping was counterbalanced across participants. A short practice block of eight trials (including some feedback about participant performance and target location) had to be completed before the two experimental blocks containing 64 trials (without feedback). Half of the trials contained a target item. A 2-min break was scheduled between experimental blocks. The two sets of potential targets (guns and knives) were presented for 10 s each at the beginning of the practice block and before the first experimental block to make participants familiar with the target items. In each trial, a fixation cross was replaced after 500 ms by an X-ray image which disappeared after 4 s. Participants had up to 20 s to respond. A blank screen of 500 ms was displayed between trials.

The main test started with written and oral instructions about the experimental condition to which participants were assigned. Participants in the diagnostic aid (DA) condition practiced with the support system during the 32 trials of the training block (50% target-present trials). During the experimental blocks, participants inspected 265 X-ray images (50% target-present trials). At the end of the experiment, participants filled in several questionnaires (i.e., trust towards automation, self-confidence in their ability to achieve the task, and subjective workload). The trial sequence was identical to the pre-test with two exceptions. First, the X-ray images stayed on screen for 20 s. Second, as mentioned above (see section ‘Simulation’), participants had to click on the ‘OK’ button if they decided there was no target in the image. If they decided there was a target item in the image, they had to mark it by clicking on it before clicking on the ‘Not OK’ (see Figure 1).

### Data Analysis

Overall, data were analyzed with a 2-way ANOVA with expertise and diagnostic aid as between-subject factors. *T*-tests for independent samples were used to compare experts and novices regarding the use of automation. One novice was excluded due to a poor detection performance in the main test (i.e., more than two SD from the mean of his/her group). With this exclusion, each experimental group contained 15 participants.

Data from the pre-test were analyzed to ensure that certified screeners had a better detection performance (d’) than novices and to examine whether participants of the same expertise level had similar performance detection before the main test. Levene’s test showed equal variances across the four groups, *F*(3,56) = 0.816, *p* = 0.491. The 2-way ANOVA confirmed that overall certified screeners performed better (*M* = 2.60, *SD* = 0.39) than novices (*M* = 1.63, *SD* = 0.41), *F*(1,56) = 87.84, *p* < 0.001, η^2^_p_ = 0.661. Furthermore, there was no main effect of diagnostic aid, *F*(1,56) = 1.014, *p* = 0.318, η^2^_p_ = 0.018, nor an interaction between expertise and diagnostic aid, *F*(1,56) = 0.002, *p* = 0.962, η^2^_p_ < 0.001. For these two effects, we used an alpha level of 0.20, following a procedure of null hypothesis testing adopted by Onnasch (2015). These results showed that participants had similar detection performance within each expertise level. Consequently, there is no need to use detection performance as a co-variate in the following analyses.

Overall, data were analyzed with a 2 × 2 ANOVA with expertise and diagnostic aid as between-subject factors. Measures collected only when the diagnostic aid was present (i.e., variables assessing use and perceived trust in automation) were analyzed with two-tailed *t*-tests. Furthermore, a correlation analysis was computed to investigate the links between perceived trust in automation and other relevant measures. Only participants who had a median LOA larger than 1 (i.e., they did not exclusively use LOA1 to solve the task) were included in this analysis.

## Results

### Performance

#### Detection Performance

**FIGURE 2:**
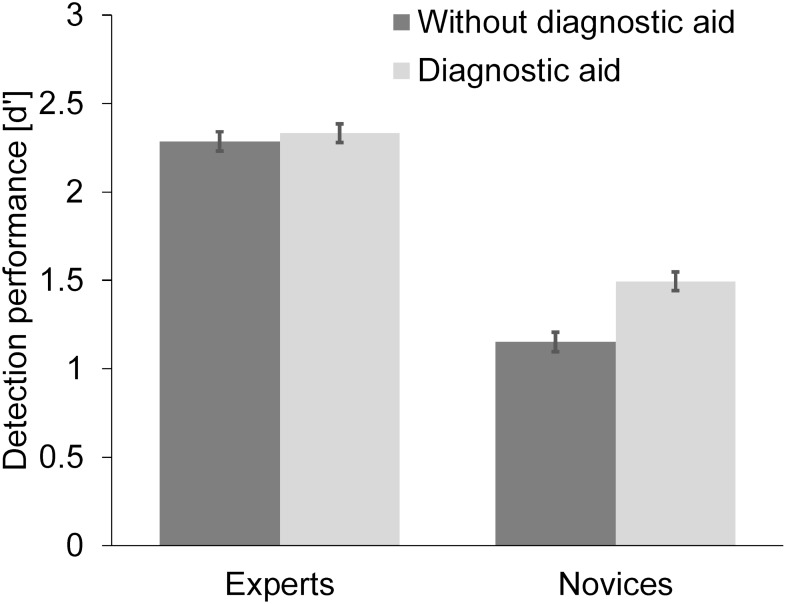
Mean detection performance as a function of expertise and presence of the diagnostic aid. Error bars correspond to the 95% confidence interval around the mean.

The 2-way ANOVA confirmed that certified screeners performed better (*M* = 2.31, *SD* = 0.29) than novices (*M* = 1.32, *SD* = 0.34, see Table 1). Furthermore, working with the diagnostic aid (DA) improved participants performance (*M*_DA_ = 1.91, *SD* = 0.65; *M_withoutDA_* = 1.81, *SD* = 0.59; see Table 1). Finally, there was a trend toward significance for the interaction between expertise and DA (see Tables 1, 2 and Figure 2). While the DA did not improve the performance of certified screeners, *t*(58) = 0.420, *p* = 0.676, *d* = 0.136, novices supported by the DA performed significantly better than those without support, *t*(58) = 3.132, *p* = 0.003, *d* = 1.132.

**Table 1:** *F*-value, significance level and effect size for the main and interaction effects for expertise and presence of the diagnostic aid.

	Expertise			Diagnostic aid			Expertise X Diagnostic aid		
			
Variable	*F*	*p*	$\eta_{p}^{2}$	*F*	*p*	$\eta_{p}^{2}$	*F*	*p*	$\eta_{p}^{2}$
*Performance*									
Detection	**162.287**	**<0.001**	**0.743**	**6.310**	**0.015**	**0.101**	3.680	0.060	0.062
Response bias	**4.817**	**0.032**	**0.079**	**14.551**	**<0.001**	**0.206**	0.180	0.673	0.003
Target localization	**173.229**	**<0.001**	**0.756**	**20.942**	**<0.001**	**0.272**	**7.398**	**0.009**	**0.117**
*Subjective measures*									
Self-confidence	**101.270**	**<0.001**	**0.644**	1.723	0.195	0.030	0.191	0.663	0.003
Perceived workload	3.463	0.068	0.058	0.000	0.985	0.000	0.092	0.762	0.002
Mental load	3.201	0.079	0.054	0.661	0.420	0.012	0.026	0.871	0.000
Physical load	0.318	0.570	0.006	0.654	0.422	0.012	1.010	0.319	0.018
Time pressure	**26.090**	**<0.001**	**0.318**	0.026	0.873	0.000	0.373	0.540	0.007
Performance	**23.345**	**<0.001**	**0.294**	0.386	0.537	0.007	1.220	0.274	0.021
Fatigue	**9.594**	**0.003**	**0.146**	0.158	0.693	0.003	0.000	1	0.000
Frustration	**4.996**	**0.029**	**0.082**	0.266	0.608	0.005	0.266	0.608	0.005


*Significant effects are marked in boldface.*

**Table 2:** Mean scores (and standard deviations) for participants’ performance use of automation, and subjective measures as a function of expertise and presence of the diagnostic aid.

	Professional screeners		Novices	
		
Score	Without DA	DA	Without DA	DA
*Performance*				
Detection [d’]	2.29 (0.30)	2.33 (0.29)	1.15 (0.31)	1.49 (0.29)
Response bias [c]	0.43 (0.19)	0.17 (0.32)	0.63 (.36)	0.30 (0.28)
Target localization [%]	74.06 (5.78)	77.81 (7.38)	41.98 (9.10)	56.72 (8.61)
*Use of automation*				
Median LOA [1–3]	-	2.23 (0.79)	-	2.67 (0.52)
LOA switches per trial	-	0.12 (0.14)	-	0.34 (0.56)
*Subjective state*				
Self-confidence [1–10]	7.73 (0.88)	7.20 (0.94)	4.53 (1.51)	4.27 (1.28)
Subjective workload [1–20]				
Mental load	13.27 (3.69)	13.80 (3.005)	14.60 (3.25)	15.40 (2.67)
Physical load	6.53 (4.98)	6.80 (6.18)	7.13 (5.10)	4.67 (4.69)
Time pressure	6.80 (4.55)	6.33 (4.15)	11.47 (3.60)	12.27 (3.69)
Performance	15.40 (2.32)	13.73 (4.01)	9.67 (3.77)	10.13 (4.50)
Fatigue	12.40 (3.29)	12.73 (4.18)	15.00 (2.75)	15.33 (2.53)
Frustration	4.93 (4.28)	4.93 (3.52)	6.93 (5.22)	8.13 (4.82)
*Trust*				
Trust perception [1–7]	-	4.27 (1.34)	-	3.95 (0.79)
Compliance [%]	-	76.06 (10.78)*	-	57.93 (11.22)
Reliance [%]	-	96.20 (3.28)*	-	88.43 (7.60)


*Note: DA = Diagnostic aid; ^∗^*n* = 12 (three participants worked only under LOA1).*

#### Criterion

Overall, certified screeners showed a significantly smaller criterion (*M* = 0.30, *SD* = 0.29) than novices (*M* = 0.47, *SD* = 0.36, see Table 1). Furthermore, working with the DA consistently reduced participants’ criterion level (*M*_DA_ = 0.24, *SD* = 0.30; *M_withoutDA_* = 0.38, *SD* = 0.33; see Table 1). Finally, the interaction between expertise and DA was not significant (see Tables 1, 2).

#### Target Localization

**FIGURE 3:**
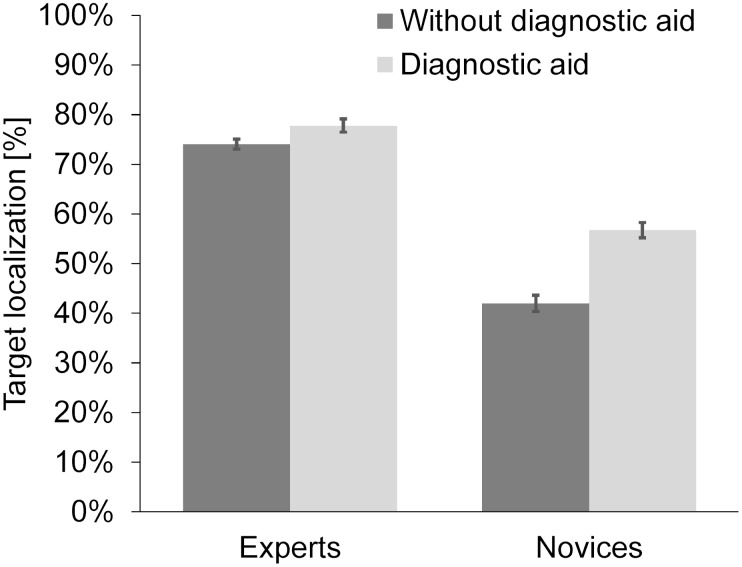
Mean percentage of correct target localization as a function of expertise and presence of the diagnostic aid. Error bars correspond to the 95% confidence interval around the mean.

Overall, in target-presence images, screeners correctly located the target item more often (*M* = 75.94 %, *SD* = 6.79) than novices (*M* = 62.64 %, *SD* = 16.35, see Table 1). Furthermore, working with the DA overall increased the percentage of correctly located targets (*M*_DA_ = 67.27 %, *SD* = 13.31; *M_withoutDA_* = 58.02%, *SD* = 17.95; see Table 1). Finally, novices profited significantly more from the presence of the DA, *t*(58) = 5.159, *p* < 0.001, *d* = 1.779, than screeners, *t*(58) = 1.312, *p* = 0.195, *d* = 0.565 (see Tables 1, 2 and Figure 3).

### Use of Diagnostic Aid

Overall, participants used a median LOA of 2.45 (SD = 0.69). Certified and novice screeners selected a similar LOA to work with during the main phase, *t*(28) = -1.772, *p* = 0.087, *d* = 0.647 (see Table 2). Furthermore, participants changed LOA 0.23 (SD = 0.41) times per trial. As for the median LOA, there was no difference between certified and novice screeners, *t*(15.623) = -1.516, *p* = 0.553, *d* = -0.553 (see Table 2).

### Subjective State

#### Self-Confidence

The two-way ANOVA revealed that certified screeners (*M* = 7.47, *SD* = 0.94) were significantly more confident in their ability to achieve the task than novices (*M* = 4.40, *SD* = 1.38; see Table 1). Neither the main effect of DA, nor its interaction with expertise were significant (see Table 1).

#### Subjective Workload

On average, participants rated their workload for this task as medium (*M* = 10.35 out of 20, *SD* = 2.27). The two-way ANOVA revealed only that the workload ratings were marginally smaller for certified screeners (*M* = 9.81, *SD* = 2.35) than novices (*M* = 10.89, *SD* = 2.10; see Table 1). To better understand where this effect came from, we analyzed each workload dimension separately with a two-way ANOVA (see Tables 1, 2) and the results were similar to those of the overall ANOVA. Certified screeners rated their mental load (*M* = 13.53, *SD* = 3.32) marginally lower than novices (*M* = 15.00, *SD* = 2.95) but there was no difference regarding the physical load (*M_Experts_* = 6.67, *SD* = 5.52; *M_Novice_* = 5.90, *SD* = 4.97). Finally, experts perceived significantly less time pressure (*M* = 6.57, *SD* = 4.29), fatigue (*M* = 12.57, *SD* = 3.70) and frustration (*M* = 4.93, *SD* = 3.85) and felt more competent (*M* = 14.57, *SD* = 3.33) than novices (time pressure: *M* = 11.87, *SD* = 3.61; fatigue: *M* = 5.17, *SD* = 2.60; frustration: *M* = 7.53, *SD* = 4.97; performance: *M* = 9.90, *SD* = 4.09).

### Trust

#### Trust Perception

Participants supported by the diagnostic aid provided on average a trust rating of 4.11 (*SD* = 1.09) on a scale from 1 to 7. There was no difference between certified and novices screeners, *t*(27.941) = 1.825, *p* = 0.079, *d* = 0.666 (see Table 2).

Regarding trust behaviors, 12 of the 15 certified screeners who worked with the DA were used in the following analyses since three of them worked only under LOA1 (i.e., they chose not to receive any support). A *t*-test showed that certified screeners had a significantly higher *compliance* rate than novices, *t*(25) = 4.245, *p* < 0.001, *d* = 1.648 (see Table 2). Furthermore, a larger *reliance* rate was observed for certified screeners than for novices, *t*(19.92) = 3.563, *p* = 0.002, *d* = 1.326 (see Table 2).

A correlational analysis was conducted between trust and different measures according to participant level of expertise (i.e., compliance, reliance, and experience expressed in months; see Table 3). It revealed that compliance is significantly correlated with trust, but only for novices. No significant correlations were observed between reliance and perceived trust neither for experts, nor novices. Finally, there was a medium (not significant) association between trust and the amount of experience.

**Table 3:** Correlation table between perceived trust and other measures as a function of expertise level (experts vs. novices).

	Perceived trust					
	
	Experts			Novices		
		
	*r*	*p*	*N*	*r*	*p*	*N*
Compliance	-0.057	0.860	12	0.559	0.030	15
Reliance	0.007	0.983	12	0.002	0.994	15
Experience	0.270	0.330	15	-		


*Only participants who had a median LOA larger than 1 were involved in those analyses.*

## Discussion

The main goal of the present study was to examine how expertise and automation influence performance, as well as use of automation and trust, in a visual inspection task. Overall, results showed that novices benefitted more from automation than experts. They achieved better detection performance when working with the support of automation than without. Despite this improvement, they could not match the performance of experts whose high detection rates were not influenced by the diagnostic aid. Furthermore, experts showed higher levels of compliance and reliance than novices. Finally, those two objective measures of trust in automation were not linked to subjective trust ratings by experts, while there was only a correlation between compliance and trust ratings by novices.

As mentioned in the introduction, visual expertise reflects complex cognitive and perceptual processing, which develop over the course of many hours of practice and training (for reviews, see Ericsson et al., 1993; Ericsson et al., 2006; Reingold and Sheridan, 2011; Sheridan and Reingold, 2017). In our study, experts achieved higher detection performance (d’) than novices. This result was expected as all screeners had been selected, qualified, trained, and certified according to the standards set by the appropriate national authority (civil aviation administration) in compliance with the relevant EU regulation (European Commission, 2015). This includes mandatory image recognition training and testing every 6 months (European Commission, 2015), which is very important for achieving and maintaining a high level of detection performance (Koller et al., 2008; Halbherr et al., 2013). Experts also felt more self-confident than novices did. This is consistent with earlier research on showing positive correlations in the range of 0.32 to 0.62 between actual performance in cognitive tasks and subjects’ confidence ratings (Shaughnessy, 1979; Koriat et al., 1980; Stankov and Crawford, 1996). While there was no effect of expertise effect on perceived physical load, experts felt more competent and they experienced less fatigue, frustration, mental load, and time pressure than novices. This could be related to findings from medical image perception, that found better detection performance of radiologists with less fixations and faster response times than novices due to holistic processing (for a recent review, see Sheridan and Reingold, 2017).

In contrast to expertise, automation had only an impact on objective measures. Novices showed better detection performance (d’) and localized targets with more accuracy when assisted by the diagnostic aid than without automated support. This pattern was not found in experts. Such an interaction was also observed in air traffic control for procedural errors (Nocera et al., 2006). Furthermore, providing participants with a diagnostic aid results in a criterion shift in both experts and novices toward a more neutral response bias. They showed less target present responses with than without DA (resulting in less false alarms but also less hits).

Unexpected results were observed for use of the DA and trust behaviors. Although both groups used a similar level of assistance throughout the entire main test, experts had different trust behaviors than novices. They were more compliant with and reliant on the diagnostic aid than novices. Interestingly, compliance rate for experts (76%) was almost identical to DA reliability when it indicated the presence of a target (75%), while reliance rate (96%) was very close to DA reliability when it reported the absence of a target (100%). Both rates for novices were lower than the actual DA reliability (58% and 88%, for compliance and reliance, respectively). This suggests that, with their experience and constant perceptual training (Halbherr et al., 2013), experts can better estimate DA performance than novices and therefore follow DA correct recommendations most of the time. This also explains why novices followed less frequently DA recommendations.

Regarding trust perception, subjective trust ratings were similar for experts and novices. However, correlations between trust perception and behaviors for experts and novices followed a similar pattern as observed for performance. Trust perception correlated with neither compliance nor reliance for experts, whereas novices with high trust ratings complied more with the DA recommendations than novices with low trust ratings. No such correlation was found for reliance. Altogether, the results of this study suggest that both experts and novices found some benefits for the presence of the DA. Experts may have used the DA as a ‘back-up’ to confirm their decision based on their expertise (confirmatory function) whereas novice may have used it as a guide to base their decisions on (support function). Even though participants were not directly asked how they used the DA, the confirmatory function for experts is indirectly supported by the positive (but not significant) correlation between trust and experience. Experienced screeners are better able to assess DA reliability and consequently displayed higher trust ratings than recently certified screeners.

This study has some limitations. The task was relatively easy to achieve for experts since only guns and knives served as prohibited items. They may have used DA in the same way as novices (i.e., support function instead of confirmatory function) if they had to detect more difficult items (i.e., explosives or shock devices). Furthermore, DA ability (i.e., how effective it is to identify an object as a prohibited item) could have changed how experts used it.

To conclude, the results of the present study suggested that expertise levels influence not how but why a DA is used. Novices seem to base their decisions on DA recommendations, whereas experts seem to confirm theirs with the DA help. Future research may use post-experimental interviews or questionnaires (e.g., about automation use or how participants estimate the reliability of the system) to test this assumption. Furthermore, it would be of considerable interest to investigate how participants use the DA when the system misses a prohibited item and what impact this would have on detection performance. Moreover, it should be investigated in future work what is the impact on DA use when the reliability level of the support system is communicated prior to the experiment. In addition, it would be of practical relevance to include X-ray images containing prohibited items which are difficult to detect even for experts (e.g., explosives, electronic shock devices, gun parts etc.).

## Ethics Statement

This study was carried out in accordance with the recommendations of the Ethics Committee of the Department of Psychology (University of Fribourg) with written informed consent from all subjects. All subjects gave written informed consent in accordance with the Declaration of Helsinki. The protocol was approved by the Ethics Committee of the Department of Psychology (University of Fribourg).

## Author Contributions

AC, AS, SM, and JS conceived and designed the study. AC performed the programming. SM and AC collected the data. AC, AS, SM, and JS wrote the manuscript.

## Conflict of Interest Statement

The authors declare that the research was conducted in the absence of any commercial or financial relationships that could be construed as a potential conflict of interest.

## References

[B1] Abadie A. Gardeazabal J. (2008). Terrorism and the world economy. *Eur. Econ. Rev.* 52 1–27. 10.1016/j.euroecorev.2007.08.005

[B2] Baum P. (2016). *Violence in the Skies: A History of Aircraft Hijacking and Bombing.* Chichester: Summersdale Publishers.

[B3] Biggs A. T. (2017). Getting satisfied with “satisfaction of search”: how to measure errors during multiple-target visual search. *Attent. Percept. Psychophys.* 79 1352–1365. 10.3758/s13414-017-1300-2 28353059

[B4] Biggs A. T. Kramer M. R. Mitroff S. R. (2018). Using cognitive psychology research to inform professional visual search operations. *J. Appl. Res. Mem. Cognit.* 7 189–198. 10.1016/j.jarmac.2018.04.001

[B5] Biggs A. T. Mitroff S. R. (2014). Different predictors of multiple-target search accuracy between nonprofessional and professional visual searchers. *Quart. J. Exp. Psychol.* 67 1335–1348. 10.1080/17470218.2013.859715 24266390

[B6] Blondon K. Wipfli R. Lovis C. (2015). “Use of eye-tracking technology in clinical reasoning: a systematic review,” in *Digital Healthcare Empowering Europeans: Proceedings of MIE2015*, eds Cornet R. Stoicu-Tivadar L. Hörbst A. Parra Calderon C. L. Andersen S. K. Hercigonja-Szekeres M. (Amsterdam: IOS Press), 90–94.

[B7] Brainard D. H. (1997). The psychophysics toolbox. *Spat. Vision* 10 433–436. 10.1163/156856897X00357 9176952

[B8] Chavaillaz A. Schwaninger A. Michel S. Sauer J. (2018). Automation in visual inspection tasks: X-ray luggage screening supported by a system of direct, indirect or adaptable cueing with low and high system reliability. *Ergonomics* 10.1080/00140139.2018.1481231 [Epub ahead of print]. 29799358

[B9] Chavaillaz A. Wastell D. Sauer J. (2016). System reliability, performance and trust in adaptable automation. *Appl. Ergon.* 52 333–342. 10.1016/j.apergo.2015.07.012 26360226

[B10] Cullen R. H. Rogers W. A. Fisk A. D. (2013). Human performance in a multiple-task environment. Effects of automation reliability on visual attention allocation. *Appl. Ergon.* 44 962–968. 10.1016/j.apergo.2013.02.010 23660082

[B11] Dzindolet M. T. Peterson S. A. Pomranky R. A. Pierce L. G. Beck H. P. (2003). The role of trust in automation reliance. *Int. J. Hum-Comput. Stud.* 58 697–718. 10.1016/S1071-5819(03)00038-7 15151155

[B12] Ericsson K. A. Charness N. Feltovich P. J. Hoffman R. R. (2006). *The Cambridge Handbook of Expertise and Expert Performance.* Cambridge: Cambridge University Press 10.1017/CBO9780511816796

[B13] Ericsson K. A. Krampe R. T. Tesch-Römer C. (1993). The role of deliberate practice in the acquisition of expert performance. *Psychol. Rev.* 100 363–406. 10.1037/0033-295X.100.3.363

[B14] European Commission (2015). Commission implementing regulation (EU) 2015/1998 laying down detailed measures for the implementation of the common basic standards on aviation security (Text with EEA relevance). *Offic. J. Eur. Union.* L 299 1–142.

[B15] Goh J. Wiegmann D. A. Madhavan P. (2005). “Effects of automation failure in a luggage screening task: a comparison between direct and indirect cueing,” in *Proceedings of the 49th Annual Meeting of the Human Factors and Ergonomics Society*, Orlando, FL, 492–496. 10.1177/154193120504900359

[B16] Green D. M. Swets J. A. (1966). *Signal Detection Theory and Psychophysics.* New-York, NY: Wiley.

[B17] Halbherr T. Schwaninger A. Budgell G. R. Wales A. W. J. (2013). Airport security screener competency. A cross-sectional and longitudinal analysis. *Int. J. Aviat. Psychol.* 23 113–129. 10.1080/10508414.2011.582455

[B18] Hardmeier D. Hofer F. Schwaninger A. (2005). “The X-ray object recognition test (X-ray ORT)-a reliable and valid instrument for measuring visual abilities needed in X-ray screening,” in *Proceeedings of the 39th IEEE Carnahan Conference on Security Technology*, Anaheim, CA, 189–192. 10.1109/CCST.2005.1594876

[B19] Harris D. H. (2002). How to really improve airport security. *Ergon. Design* 10 17–22. 10.1177/106480460201000104

[B20] Hart S. G. Staveland L. E. (1988). “Development of NASA-TLX (Task Load Index): results of empirical and theoretical research,” in *Human Mental Workload*, eds Hancock P. A. Meshkati N. (Amsterdam: North-Holland), 139–183.

[B21] Hättenschwiler N. Sterchi Y. Mendes M. Schwaninger A. (2018). Automation in airport security X-ray screening of cabin baggage: examining benefits and possible implementations of automated explosives detection. *Appl. Ergon.* 72 58–68. 10.1016/j.apergo.2018.05.003 29885728

[B22] Hoffman R. R. Johnson M. Bradshaw J. M. Underbrink A. (2013). Trust in automation. *IEEE Intell. Syst.* 28 84–88. 10.1109/MIS.2013.24

[B23] Hoffman R. R. Ward P. Feltovich P. J. DiBello L. Fiore S. M. Andrews D. H. (2014). *Accelerated Learning: Training for High Proficiency in a Complex World.* Hoboken, NJ: Taylor and Francis.

[B24] Jian J.-Y. Bisantz A. M. Drury C. G. (2000). Foundations for an empirically determined scale of trust in automated systems. *Int. J. Cogn. Ergon.* 4 53–71. 10.1207/S15327566IJCE0401_04

[B25] Jipp M. (2016). Expertise development with different types of automation. *Hum Fact.* 58 92–106. 10.1177/0018720815604441 26407588

[B26] Kleiner M. Brainard D. H. Pelli D. G. Ingling A. Murray R. Broussard C. (2007). What’s new in psychtoolbox-3. *Perception* 36:1.

[B27] Koller S. M. Drury C. G. Schwaninger A. (2009). Change of search time and non-search time in X-ray baggage screening due to training. *Ergonomics* 52 644–656. 10.1080/00140130802526935 19424926

[B28] Koller S. M. Hardmeier D. Michel S. Schwaninger A. (2008). Investigating training, transfer and viewpoint effects resulting from recurrent CBT of X-Ray image interpretation. *J. Trans. Sec.* 1 81–106. 10.1007/s12198-007-0006-4

[B29] Koriat A. Lichtenstein S. Fischhoff B. (1980). Reasons for confidence. *J. Exp. Psychol. Hum. Learn. Mem.* 6 107–118. 10.1037/0278-7393.6.2.107

[B30] Lee J. D. Moray N. (1992). Trust, control strategies and allocation of function in human-machine systems. *Ergonomics* 35 1243–1270. 10.1080/00140139208967392 1516577

[B31] Lee J. D. See K. A. (2004). Trust in automation. Designing for appropriate reliance. *Hum. Fact.* 46 50–80. 10.1518/hfes.46.1.50_30392 15151155

[B32] Lehr P. (2019). “Detection: scanning and ‘sniffing’ technologies,” in *Counter-Terrorism Technologies: A Critical Assessment*, ed. Lehr P. (Cham: Springer International Publishing), 101–114. 10.1007/978-3-319-90924-0_7

[B33] Litchfield D. Donovan T. (2017). The flash-preview moving window paradigm. Unpacking visual expertise one glimpse at a time. *Front. Learn. Res.* 5 66–80. 10.14786/flr.v5i3.269

[B34] Macmillan N. A. Creelman C. D. (2005). *Detection Theory: A User’s Guide.* 1st Edn. Mahwah, NJ: Psychology Press.

[B35] Mery D. Mondragon G. Riffo V. Zuccar I. (2013). Detection of regular objects in baggage using multiple X-ray views. *Insight* 55 16–20. 10.1784/insi.2012.55.1.16

[B36] Meyer J. (2001). Effects of warning validity and proximity on responses to warnings. *Hum. Fact.* 43 563–572. 10.1518/001872001775870395 12002005

[B37] Michel S. Koller S. M. Ruiter J. C. de Moerland R. Hogervorst M. Schwaninger A. (2007). “Computer-based training increases efficiency in X-ray image interpretation by aviation security screeners,” in *Proceedings of th 41st Annual IEEE International Carnahan Conference on Security Technology*, ed. Sanson L. D. (Piscataway, NJ: IEEE), 201–206. 10.1109/CCST.2007.4373490

[B38] Mitroff S. R. Biggs A. T. Cain M. S. (2015). Multiple-target visual search errors. *Policy Insights Behav. Brain Sci.* 2 121–128. 10.1177/2372732215601111

[B39] Nocera F. Di Fabrizi R. Terenzi M. Ferlazzo F. (2006). Procedural errors in air traffic control: effects of traffic density, expertise, and automation. *Aviat. Space Environ. Med.* 77 639–643. 16780243

[B40] Onnasch L. (2015). Crossing the boundaries of automation-function allocation and reliability. *Int. J. Hum. Comput. Stud.* 76 12–21. 10.1016/j.ijhcs.2014.12.004

[B41] Parasuraman R. Hancock P. A. (2008). “Mitigating the adverse effects of workload, stress, and fatigue with adaptive automation,” in *Performance Under Stress (Human Factors in Defence)*, eds Szalma J. L. Hancock P. A. (Aldershot: Ashgate), 45–58.

[B42] Pelli D. G. (1997). The videotoolbox software for visual psychophysics: transforming numbers into movies. *Spat. Vis.* 10 437–442. 10.1163/156856897X00366 9176953

[B43] Reingold E. M. Sheridan H. (2011). “Eye movements and visual expertise in chess and medicine,” in *The Oxford Handbook of Eye Movements*, 1st Edn, eds Liversedge S. P. Gilchrist I. D. Everling S. (Oxford: Oxford University Press), 523–550.

[B44] Rice S. McCarley J. S. (2011). Effects of response bias and judgment framing on operator use of an automated aid in a target detection task. *J. Exp. Psychol. Appl.* 17 320–331. 10.1037/a0024243 21707202

[B45] Rovira E. McGarry K. Parasuraman R. (2007). Effects of imperfect automation on decision making in a simulated command and control task. *Hum. Fact.* 49 76–87. 10.1518/001872007779598082 17315845

[B46] Schuster D. Rivera J. Sellers B. C. Fiore S. M. Jentsch F. (2013). Perceptual training for visual search. *Ergonomics* 56 1101–1115. 10.1080/00140139.2013.790481 23650877

[B47] Schwaninger A. (2005). Increasing efficiency in airport security screening. *WIT Trans. Built Environ.* 82 407–416.

[B48] Schwaninger A. Hardmeier D. Hofer F. (2005). Aviation security screeners visual abilities & visual knowledge measurement. *Aerospace Electron. Syst. Mag. IEEE* 20 29–35.

[B49] Shanteau J. (1992). “The psychology of experts: an alternative view,” in *Expertise and Decision Support*, eds Wright G. Bolger F. (Boston, MA: Springer US), 11–23. 10.1007/978-0-585-34290-0_2

[B50] Shaughnessy J. J. (1979). Confidence-judgment accuracy as a predictor of test performance. *J. Res. Personal.* 13 505–514. 10.1016/0092-6566(79)90012-6

[B51] Sheridan H. Reingold E. M. (2017). The holistic processing account of visual expertise in medical image perception: a review. *Front. Psychol.* 8:323. 10.3389/fpsyg.2017.01620 29033865 PMC5627012

[B52] Stankov L. Crawford J. D. (1996). Confidence judgments in studies of individual differences. *Personal. Ind. Diff.* 21 971–986. 10.1016/S0191-8869(96)00130-4

[B53] Sterchi Y. Schwaninger A. (2015). “A first simulation on optimizing EDS for cabin baggage screening regarding throughput,” in *International Carnahan Conference on Security Technology (ICCST)* (Piscataway, NJ: IEEE), 55–60. 10.1109/CCST.2015.7389657

[B54] Wales A. W. J. Anderson C. Jones K. L. Schwaninger A. Horne J. A. (2009). Evaluating the two-component inspection model in a simplified luggage search task. *Behav. Res. Methods* 41 937–943. 10.3758/BRM.41.3.937 19587210

[B55] Wells K. Bradley D. A. (2012). A review of X-ray explosives detection techniques for checked baggage. *Appl. Radiat. Isotopes* 70 1729–1746. 10.1016/j.apradiso.2012.01.011 22608981

[B56] Wickens C. D. Dixon S. R. (2007). The benefits of imperfect diagnostic automation: a synthesis of the literature. *Theor. Issues Ergon. Sci.* 8 201–212. 10.1080/14639220500370105

[B57] Wiegmann D. A. McCarley J. S. Kramer A. F. Wickens C. D. (2006). Age et automation interact to influence performance of a simulated luggage screening task. *Aviat. Space Environ. Med.* 77 825–831. 16909876

[B58] Wolfe J. M. Evans K. K. Drew T. Aizenman A. Josephs E. (2016). How do radiologists use the human search engine? *Radiat. Prot. Dosim.* 169 24–31. 10.1093/rpd/ncv501 26656078 PMC4911962

[B59] Wolfe J. M. Horowitz T. S. van Wert M. J. Kenner N. M. Place S. S. Kibbi N. (2007). Low target prevalence is a stubborn source of errors in visual search tasks. *J. Exp. Psychol. Gen.* 136:623. 10.1037/0096-3445.136.4.623 17999575 PMC2662480

[B60] Wolfe J. M. van Wert M. J. (2010). Varying target prevalence reveals two dissociable decision criteria in visual search. *Curr. Biol.* 20 121–124. 10.1016/j.cub.2009.11.066 20079642 PMC2818748

[B61] Zuboff S. (1998). *In the Age of the Smart Machine: The Future of Work and Power.* New-York, NY: Basic Books.

